# Anti-ulcer activity of green synthesized selenium nanoparticles using *Nigella sativa* L. seed extract in rats

**DOI:** 10.3389/fphar.2025.1638593

**Published:** 2025-11-21

**Authors:** Awatef Elwej, Fawziah Al-Otaibi, Khadija Boukholda, Fahad Alminderej, Faez Alotaibi, Amani Kaabi, Nada M. Ali, Hamadi Fetoui, Kaiss Aouadi, Adel Kadri

**Affiliations:** 1 Laboratory of Toxicology and Environmental Health, LR17ES06, Sciences Faculty of Sfax, University of Sfax, Sfax, Tunisia; 2 Research Laboratory on Energy and Matter for Nuclear Science Development (LR16CNSTN02), National Center for Nuclear Sciences and Technologies, Tunis, Tunisia; 3 Department of Chemistry, College of Science, Qassim University, Buraydah, Saudi Arabia; 4 Department of Neurobiology, Center for Neurotrauma, Multiomics & Biomarkers, Morehouse School of Medicine, Atlanta, GA, United States; 5 Chemistry Department, Faculty of Science, Al-Baha University, Al-Baha, Saudi Arabia

**Keywords:** green synthesis, selenium nanoparticles, gastric ulcer, oxidative Stress, *Nigella sativa L. seed*, rats

## Abstract

**Background/Objectives:**

Selenium nanoparticles (SeNPs) have gained importance due to their potential biological properties. The present work is the first study to investigate the protective effect of selenium nanoparticles (SeNPs) synthesized using *Nigella sativa* L. seed extract on stomach ulcers in adult rats.

**Methods:**

The formation of SeNPs was confirmed by Ultraviolet–visible (UV), Fourier Transform Infrared Spectroscopy (IR), Thermal-gravimetric analysis (TGA-DTA coupled system), X-ray diffraction analysis (XRD) and scanning electron microscope (SEM) analysis. Animals were classified into 4 distinct groups. Group 1, serving as controls; group 2, serving as ulcer-group where rats received a single oral dosage of 96% ethanol (5 mL/Kg BW). Rats in Group 3 were given orally 0.5 mg/kg BW of SeNPs 1 hour before ethanol-induced gastric ulcer. Group 4 received SeNPs only (0.5 mg/kg BW) by intragastric way and served as a positive control.

**Results:**

Green synthesis was confirmed via UV-Vis spectroscopy (230 nm peak), FTIR revealed functional groups (O–H, C=O, Se–O or Se–Se). XRD pattern shows an average crystallite sizes of nanoparticles were around 26 (4) and 268 (4) nm for β-SeO2 and g-SeO2 forms, respectively. SEM examination indicated that SeNPs have a predominantly spherical to sub-spherical morphology. TG-DTA analysis demonstrates the good thermal stability of selenium nanoparticles, evidenced by initial moisture loss, controlled degradation of organic stabilizers and the formation of a stable inorganic selenium core. SeNPs’ protective effects were assessed by evaluating the ulcer index, conducting histological analysis, measuring oxidative stress markers and antioxidant defenses, as well as examining key factors involved in gastric mucosal protection. Our results demonstrated that SeNPs reduced malondialdehyde (MDA), and advanced oxidation protein product (AOPP) levels, nitric oxide (NO) levels in stomach of ethanol-induced gastric ulcer, as well as the activities of Catalase (CAT), glutathione peroxidase (GPx) and superoxide dismutase (SOD); while glutathione (GSH) and non-protein thiols (NPSH) levels were restored reaching control values. Moreover, the gastric healing effect of SeNPs pretreatment was associated with an improvement in hematological parameters and a reduction in CRP levels.

**Conclusion:**

These findings underscore the potential of SeNPs to enhance the antioxidant defense system of gastric mucosal cells and prevent ethanol-induced gastric damage in rats.

## 1 Introduction

Gastric ulcers are a serious gastrointestinal condition that occurred when there is a disruption in the balance between the stomach’s protective mechanisms, known as the gastric mucosal barrier, and various damaging factors (Khan et al., 2024; Shaik, 2024). The mucosal lining of the stomach is particularly sensitive to various harmful factors such as excessive alcohol intake, frequent use of nonsteroidal anti-inflammatory drugs (NSAIDs), chronic stress, and irregular dietary habits (Choi et al., 2024). These factors lead to drastic stomach alterations and eventually to the development of gastric ulcers. Given the severity of these complications, researchers have increasingly focused on the development of new gastro-protective medications from both synthetic and natural sources (Uthansingh et al., 2024; Yadav et al., 2024).

Recently, treatments by based-herbal are currently receiving more attention, particularly nanoparticles loaded-plant (Sharifi-Rad et al., 2020; Shoman et al., 2025). Nanoparticles (NP) have gained significant attention in the pharmaceutical industry due to their advantageous properties (Sharifi-Rad et al., 2024; El Messaoudi et al., 2025; Mirzaei et al., 2025). NP-based drug delivery systems can effectively address pharmacokinetic limitations and enhance the efficacy of conventional therapeutic agents. Moreover, these nanoparticle formulations facilitate rapid entry into the bloodstream, prolonged circulation time, improved bioavailability (Dewangan et al., 2022), and reduced adverse effects of medications. Selenium is an essential trace element that exhibits both antioxidative and prooxidative properties depending on its concentration and bioavailability (Islam et al., 2023; Pyrzynska and Sentkowska, 2024; Wang et al., 2024). It can act as a co-factor for various antioxidant enzymes. Selenium interacts with selenoproteins to exert antioxidant, anti-inflammatory, antiviral, and antitumor effects within the body (Rua et al., 2023). SeNPs have garnered interest due to their excellent bioavailability, low toxicity, and unique biological activities (Zambonino et al., 2023; El Messaoudi et al., 2025). The antioxidant properties of selenium nanoparticles stem from their ability to enhance the body’s natural defense against oxidative stress, boost the activity of key antioxidant selenoenzymes like glutathione peroxidase, which neutralize harmful reactive oxygen species (ROS). Additionally, SeNPs can directly scavenge free radicals, thereby reduce inflammation and protect organs from damage. Their nanoscale size improves bioavailability and efficacy, making them potent antioxidants with low toxicity and high biocompatibility. In recent work, researchers demonstrated that SeNPs can mitigate oxidative stress induced by toxic substances like chromium the activities of SOD, CAT, and glutathione levels in treated animals (Karas et al., 2024; Mumtaz et al., 2024). Additionally, SeNPs have shown potential in alleviating neurotoxicity and nephrotoxicity caused by cadmium chloride through the reduction of oxidative stress. The protective effects of various nanoparticles in minimizing drug-induced side effects are promising. Recent studies have explored the utilization of nanoparticles for delivering therapeutic agents like *Nigella sativa* (Ambreen et al., 2024; Daoudi et al., 2024; Hadian et al., 2024). An environmentally responsible way to produce nanoparticles is using green synthesis. Due to its well-known therapeutic effects, *Nigella sativa L.*—belonging from Renunculaceae family—is a plant that shows great attention and use in different civilizations. Its seeds represent the main useful and nutraceutical product for the treatment of such diseases related to kidney and liver function, circulatory and immune system, respiratory, intestinal and stomach diseases, and for the general wellbeing (Ben Amara et al., 2025).

Our study addresses a significant research need by exploring, for the first time, the combined use of *Nigella sativa* L. seed extract and selenium for the green synthesis of SeNPs aimed at protecting and healing gastric ulcers. Although previous research has demonstrated the gastroprotective and antioxidant properties of *Nigella sativa* L. and SeNPs, separately, as well as the ability of *Nigella sativa* L. seed extracts to enhance drug accumulation and reduce toxicity (Hannan et al., 2021; Nauroze et al., 2023), their combined effects have not yet been investigated.

In this study, we synthesize SeNPs using *Nigella sativa* L. seed extract and evaluate their protective effects against ethanol-induced gastric ulcers in adult rats. We hypothesize that this novel, green nanotechnology approach will yield nanoparticles with enhanced antioxidant activity, improved biocompatibility, and reduced toxicity, thereby offering a safer and more effective therapeutic strategy for the prevention and treatment of gastric ulcers.

## 2 Materials and methods

### 2.1 Drug and chemicals

*Nigella sativa* L. [Ranunculaceae; *Nigellae sativae semen*], commonly called black cumin and “sinouj” in Tunisia, is an annual herbaceous plant cultivated in different parts of the world, mainly in countries bordering the Mediterranean Sea. *Nigella sativa* L. seeds were purchased from local area in Sfax (Tunisia). This plant was botanically identified by Pr Khalil Mseddi, Professor of Vegetal Biology in Science Faculty of Sfax, Tunisia. A voucher specimen (No. NS-2025) was deposited in the herbarium of the biology department, Science Faculty of Sfax.

All other chemicals were acquired from commercial suppliers.

### 2.2 *Nigella sativa* L. seed extract preparation

The *Nigella sativa* L. seeds were first washed several times then they were dried at room temperature. After the seeds were allowed to dry for a specified duration, they were ground into a fine powder and dissolved in distilled water. The drug-extract ratio (DER) for our preparation was 1:5 (w/v). The mixture was continuously stirred and heated (60 °C), for 30 min under constant agitation (Waseem et al., 2024). Finally, the extract of *Nigella sativa* L. seeds was collected and filtered in Whatman No. 1 to be stored (4 °C) in dark for further use in the nanoparticle synthesis without any further concentration standardization.

### 2.3 Green synthesis of SeNPs

The biosynthesis of SeNPs using *Nigella sativa* L. seed extract was outlined according to (Ahmad et al., 2024) with slight modification. Briefly, 2.5 mL of 10 mM Na_2_SeO_3_ was added to 20 mL of N. Sativa seed extract and the mixture was mixed for 5 min in a magnetic stirrer. Then, 1 mL of aqueous ascorbic acid solution (40 mM) was added dropwise into the resulting mixture under magnetic stirring at 80 °C for approximately 4 h at pH 9. The resulting complex was collected after centrifugation at 10,000 rpm for 10 min, washed with water and centrifuged again at 5,000 rpm for 10 min. The separated complex was dried in an oven at 40 °C for 8 h. After drying, SeNPs were scratched and stored in Eppendorf tube for further use.

### 2.4 Characterization of SeNPs

UV–VIS spectra was used to follow the absorption spectrum measurements of the formed SeNPs in the extract of *Nigella sativa* L. seeds which were read in the range of 200–800 using a UV–visible spectrophotometer (GENESYS 50, UV-2600). The Fourier transform infrared (FT-IR) spectra of SeNPs samples (1–2 mg) was recorded at the scanning range of 400–4,000 cm^−1^ using Vetex70 (Brock, Germany).

### 2.5 XRD, SEM and TGA-DTA analysis of SeNPs

XRD analysis was carried out on the selenium nanocomposite to study their crystalline structure and phase composition. Sample was examined using an X’Pert Philips diffractometer, which operated with CuKα radiation (λ = 1.54 Å) at 40 kV. The surface morphology and particle shape of the selenium nanocomposites were characterized using a JEOL JSM-6510LV scanning electron microscope (SEM) (JEOL Ltd., Japan). Thermal stability and decomposition behavior were investigated by thermal analysis with a TGA-DTA coupled system (Mettler/Toledo). The experiments were conducted at a heating rate of 10 °C/min under a nitrogen atmosphere, with a temperature precision of ±0.3 K, ensuring accuracy and reproducibility of the thermal measurements.

### 2.6 Animals and experimental design

#### 2.6.1 Animals

Central Pharmacy (SIPHAT, Tunisia) provided Female Wistar rats (180–200 g) which were acclimatized in a room with relative humidity and temperature of 40% and 22 °C ± 2 °C, under a 12-h light-dark phase. All of the rats were treated in conformity with the General Guidelines on the Use of living Animals in Scientific Investigations (1986) and approved by the Committee for the Care and Use of Laboratory Animals.

#### 2.6.2 Ethanol-induced gastric mucosal injury in rats

**FIGURE 1:**
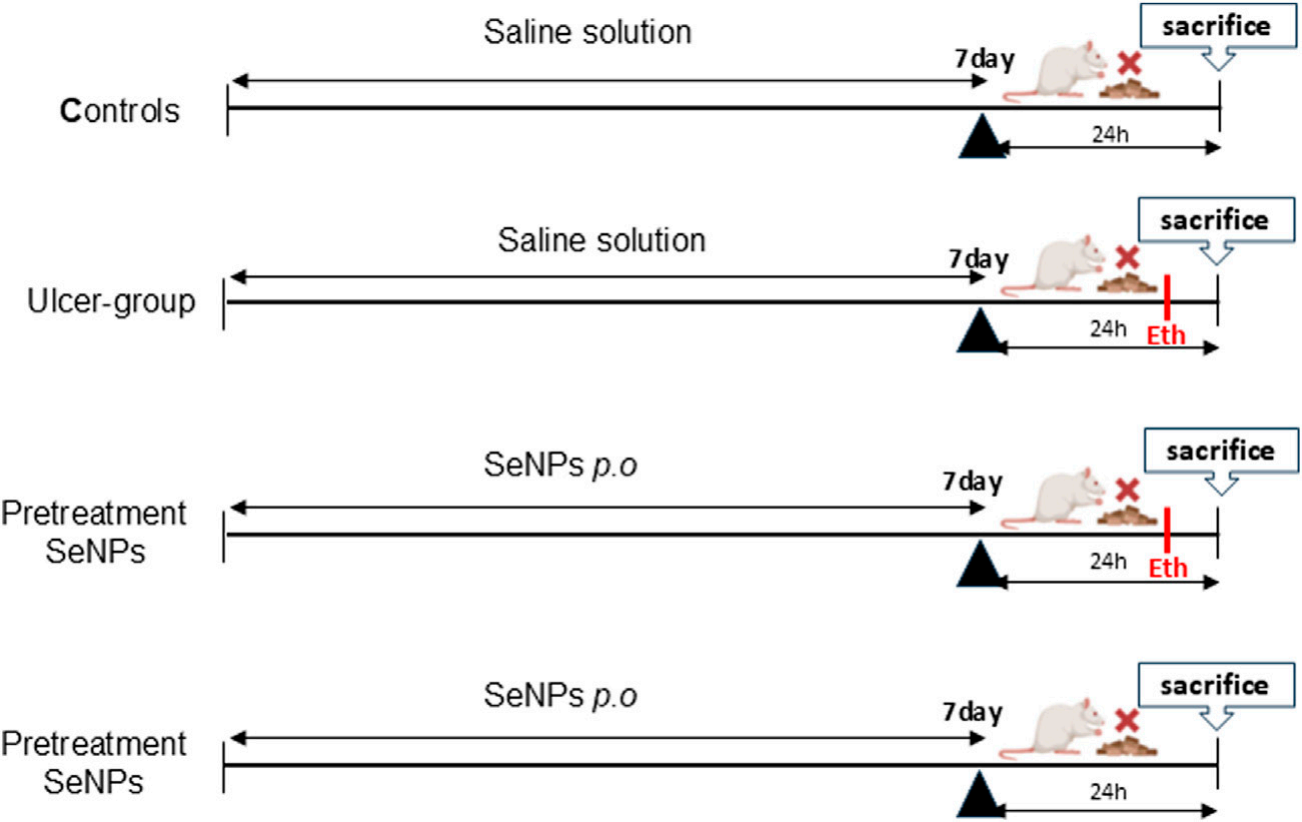
Protocol design.

The Wistar rats were randomly assigned to five groups each consisting of 6 animals (1) control, (2) model (ethanolic group = Eth), (3) SeNPs-Eth (0.5 mg.kg^−1^), 4 SeNPs (0.5 mg.kg^−1^) as positive group. The intragastric gavage of SeNPs used for all treatments lasted for 7 days. At the end of the experiment (7 days) and 1 hour after SeNPs administration, 1 mL of 96% ethanol (Beiranvand and Bahramikia, 2020; Araújo et al., 2024) was administered per gavage to induce gastric ulcer, in the second and third groups. In order to avoid coprophagy, which obstructs the development of stomach ulcers, all the animals were not provided with food but given unrestricted access to water in metabolic cages for 24 h prior to sacrifice. All the rats were decapitated after 1 hour of ulcer induction (Figure 1). Before taking pictures, their stomachs were taken out and divided along the larger curvature. They were then put on a white paper to measure the length of the ulcers and rapidly washed with cold saline solution. Each tissue was then dichotomized. For histological assessment, one moiety was preserved in 10% formaldehyde, while the other was kept at −80 °C for further examination.

#### 2.6.3 Hematological parameters and C-Reactive protein (CRP) assessment

All hematological parameters and C-Reactive Protein (CRP) were analyzed by electronic automate coulter MAXM (Beckman Coulter, Inc., Fullerton, CA).

#### 2.6.4 Stomach ulcer index (UI) and inhibitory rate (%) determination

Stomach ulcer index (UI) (Equation 1), expressed in square millimeters (mm2), was determined using (Aliouche et al., 2024)’s method to assess the severity of the observed red injury bands. These were evaluated under magnification in a blinded manner and graded as follow: [0] = no lesion; [1] was awarded for erosion lengths less to 1 mm, [2] for erosion lengths between 1 and 2 mm, [3] for erosion lengths between 2 and 4 mm. Random measurements of ulcer index scoring were performed to assess intra-observer consistency and inter-observer reliability improving the accuracy of the results.

The following calculation was used to calculate ulceration protection (Equation 2):

$$\text{Ulcer index }\left(\text{UI}\right)=\frac{\text{Total}\;\text{degrees}\;\text{of}\;\text{Ulcers}\;\text{in}\;\text{each}\;\text{group}}{\text{Number}\;\text{of}\;\text{animals}\;\text{in}\;\text{each}\;\text{Ulcer}\;}$$ (1)

The percentage of ulcer inhibition rate was calculated as below:

$$\text{Protection of ulceration }\left(I\%\right)=\frac{\left(\text{ulcer}\;\text{index}\;\text{in}\;\text{the}\;\text{model}\;\text{group}-\text{ulcer}\;\text{index}\;\text{in}\;\text{group}\;3\right)}{\text{ulcer}\;\text{index}\;\text{in}\;\text{the}\;\text{model}\;\text{group}}x100$$ (2)

#### 2.6.5 Determination of the related biochemical indexes in gastric tissues

The stomach samples were weighed, homogenized in ice cold saline, and split into two groups. One part was centrifuged at 4,000 rpm for 10 min, and the supernatant was used to measure MDA, AOPP, and NO levels, as well as SOD and CAT and GPx activity.

##### 2.6.5.1 Nitric oxide (NO) level assay

Nitric oxide (NO) levels in liver tissue were measured using the colorimetric Griess reaction, with quantification based on absorbance at 540 nm [24].

##### 2.6.5.2 Evaluation of stomach tissue lipid peroxidation

Gastric lipid peroxidation was measured using a colorimetric assay for thiobarbituric acid reactive substances (TBARS), with results expressed as malondialdehyde (MDA) concentrations, following the method of Draper and Hadley [25]. MDA levels were reported in nanomoles per milligram of protein.

##### 2.6.5.3 Advanced oxidation protein products (AOPP) content

As common markers of protein oxidation, the levels of advanced oxidation protein products was measured in stomach homogenate tissues (Turkyilmaz et al., 2021) to identify the level of oxidative stress. Briefly, the stomach AOPP level was determined spectrophotometrically, and the absorbance of each sample was measured at 340 nm using the extinction coefficient of 26,000 M^−1^ cm^−1^.

##### 2.6.5.4 Evaluation of stomach non-enzymatic oxidative stress markers and antioxidant enzymatic activities

Stomach glutathione (GSH), Non-protein thiols (NPSH) stomach tissue levels, and the SOD*,* the CAT and the GPx activities were determined.

#### 2.6.6 Histological assessment of gastric tissue

Small sections of stomach tissue were preserved and prepared. Histological assessment was performed using five micrometer-thick sections stained with hematoxylin and eosin (H&E). The severity of stomach histology scores was determined using scores on a scale of none (−), mild (+), moderate (++) and severe (+++) damages.

### 2.7 Statistical analysis

Data was analyzed with GraphPad Prism 10.4.0 and presented as mean ± SEM. One-way ANOVA with Tukey’s *post hoc* test was used for statistical analysis.

## 3 Results

### 3.1 UV–visible and IR analyses

**FIGURE 2:**
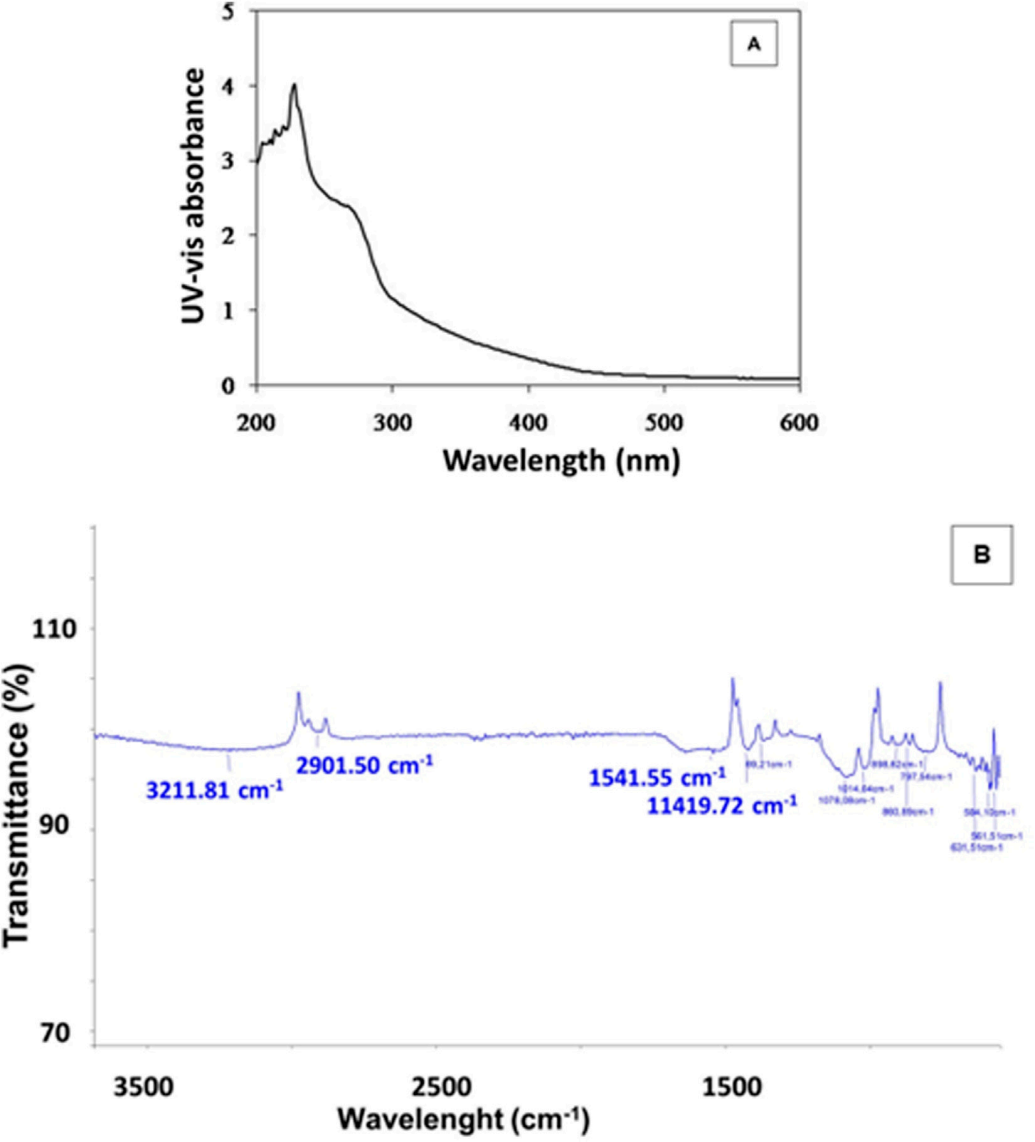
UV-vis spectrum **(A)** and FTIR analysis **(B)** of SeNPs mediated by *Nigella sativa L.* seed extract.

The formation of selenium nanoparticles (SeNPs) from *Nigella sativa* L. seed extract was primarily confirmed by UV–Visible spectroscopy at the wavelengths between 200 and 800 nm. The absorption spectrum displayed a characteristic peak around 230 nm, attributed to the surface plasmon resonance of the biosynthesized SeNPs, indicating their successful formation and nanoscale dimension (Figure 2A). This optical behavior is consistent with previous reports on green-synthesized selenium nanoparticles and suggests stable colloidal dispersion and various locations for the absorption peak (Pyrzynska, 2024; Stella et al., 2025). Further insight into the functional groups involved in the reduction and stabilization of SeNPs was provided by FTIR analysis (Figure 2B). The spectrum revealed a broad absorption band near 3,211 cm^−1^, corresponding to O–H stretching vibrations of hydroxyl groups from phenolic compounds present in the *Nigella sativa* L. seed extract. This peak aligns well with reported FTIR spectra of selenium nanoparticles, where O–H stretching vibrations typically appear between 3,200 and 3,300 cm^−1^, indicating the presence of hydroxyl and phenolic functional groups that often cap or stabilize the nanoparticles. Similar findings have been reported in green-synthesized selenium nanoparticles characterized by FTIR, confirming these characteristic absorption bands associated with surface functional groups (Alvi et al., 2021). A distinct peak at 1,541 cm^−1^ was assigned to the C=O stretching of amide or carbonyl groups, indicating the involvement of proteins or other bio-organic molecules in the stabilization of selenium nanoparticles. This observation is consistent with recent reports where reports where amide II absorption bands around 1,540 cm^−1^ was observed in selenium nanoparticles, confirming the role of biomolecules as capping agents (Kamnev et al., 2021). Additional peaks observed around 1,400 cm^−1^ and 1,100 cm^−1^ were attributed to C–H bending and C–O stretching vibrations, respectively. Our findings are consistent with previous reports on selenium nanoparticles where peaks near 1,400 cm^−1^ correspond to methyl or methylene C–H bending vibrations, and peaks around 1,100 cm^−1^ are commonly linked to C–O stretching vibrations of phenolic compounds involved in nanoparticle capping and stabilization (Ahmed et al., 2025). A weak band near 631 cm^−1^ may be associated with Se–O or Se–Se bond vibrations, supporting the presence of elemental selenium. Similar absorption bands have been reported in FTIR spectra of selenium nanoparticles, indicating characteristic vibrations of selenium-selenium or selenium-oxygen bonds related to the elemental or oxidized selenium species (El-Batal et al., 2021). These findings collectively confirm that selenium compounds not only reduce selenium ions but also stabilize the formed nanoparticles, facilitating controlled synthesis and enhanced nanoparticle stability. This dual role has been reported in several studies investigating selenium nanoparticle synthesis and stabilization mechanisms.

### 3.2 XRD analysis

**FIGURE 3:**
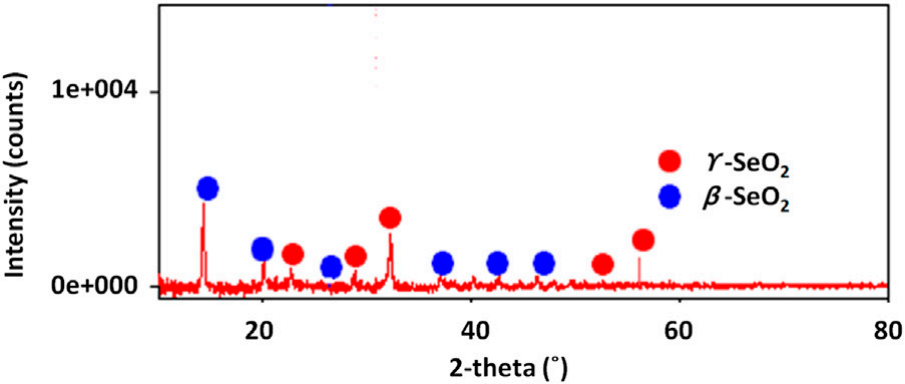
XRD diffractogram of selenium nanoparticles synthesized from *Nigella sativa L.* seed extract.

Figure 3 shows the result of the XRD analysis of selenium nanoparticles synthesized from *Nigella sativa* L. *seed* extract. Indexing of the different diffraction peaks reveals the presence of two forms of selenium oxides (*β* and *γ*), suggesting that the Se nanoparticles have partially oxidized (formation of SeO_2_ or other oxidized phases) either during synthesis, or during drying and/or storage. SeO_2_ has several polymorphs, and *β/γ* phases have been described experimentally (Orosel et al., 2004). It is common, in so-called “green” synthesis (plant extract), for a fraction of the selenium to remain in oxidized form, either because the reducing power of the extract is insufficient, or because there is reoxidation in air. The refined structure of the present selenium nanoparticles coincides with the orthorombic structure with space group Pmc21 and lattice parameters a = 5.082 (1), b = 4.522 (2), and c = 7.658 Å for the form *β* and a = 5.036 (1), b = 4.63 (2), and c = 14.64 (3) Å for the form *γ* (Orosel et al., 2004).

The crystallite sizes (D) of the selenium nanoparticles in their two identified *β* and *γ* forms were measured using Scherer’s relation (Khitouni, 2003):

$$D=\frac{k.\lambda }{\beta \cos\theta }$$ (3)

where k is a constant (k = 0.94), λ represents the XRD wavelengths, β is the line width at the half-maximum of the peak intensity, and θ is the diffraction angle. The estimated average crystallite sizes of nanoparticles were around 26 (4) and 268 (4) nm for *β*-SeO2 and γ-SeO2 forms, respectively.

### 3.3 SEM analysis

**FIGURE 4:**
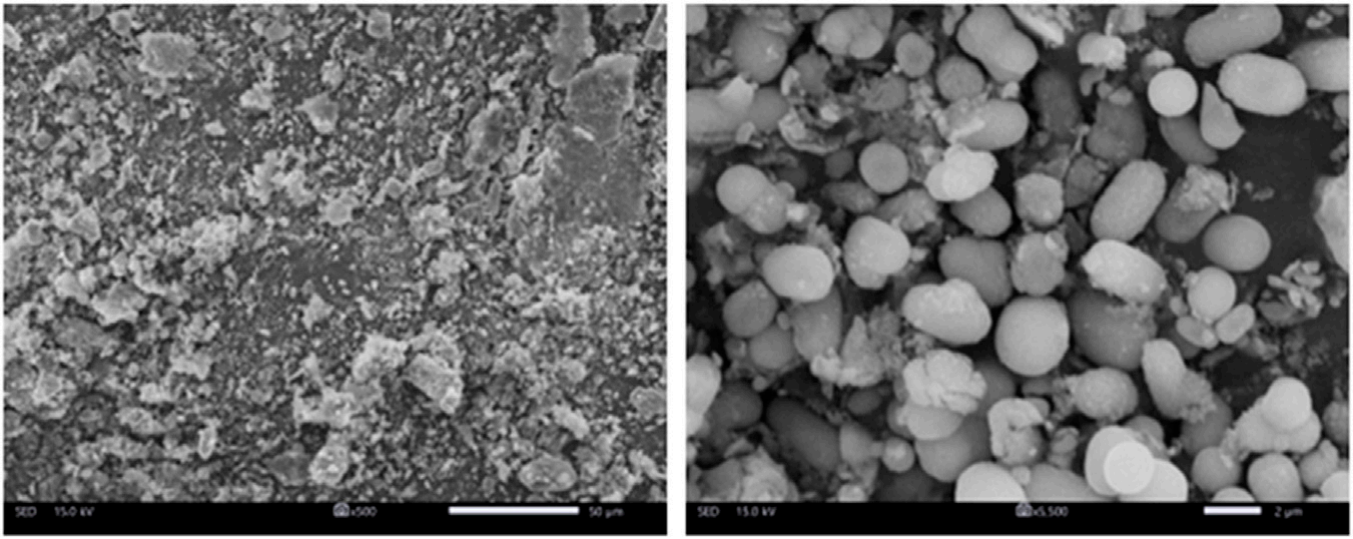
SEM images of selenium nanoparticles obtained from *Nigella sativa L.* seed extract for low and high magnification (500x and 5.500x).

SEM analysis shows that selenium nanoparticles obtained from *Nigella sativa* L. seed extract have a predominantly spherical to sub-spherical morphology (Figure 4). The particles appear generally well-formed, but with a marked tendency to agglomerate, leading to the formation of more or less compact clusters. The isolated particles observed display nanometric dimensions mainly between 100 and 300 nm, with an average size close to 200 nm. In contrast, the inclusion of agglomerated particles in the analysis reveals a broader size distribution and increased polydispersity, with some entities reaching several hundred nanometers. This morphology confirms the role of the plant extract as a reducing and stabilizing agent, allowing the nucleation and growth of spherical nanoparticles, while allowing interparticle interactions responsible for agglutination to persist.

### 3.4 TG-DTA analyses

**FIGURE 5:**
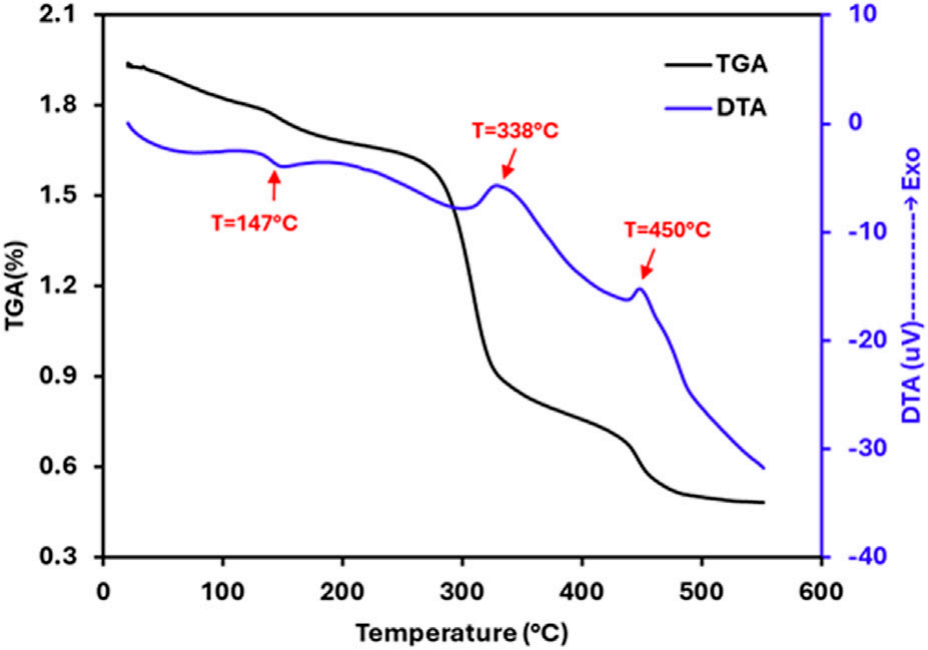
TGA-DTA curves of the selenium nanoparticles synthesized from *Nigella sativa L.* seed extract.

The thermal features of the selenium nanoparticles synthesized from *Nigella sativa* L. seed extract were studied by using a TGA-DTA coupled instrument. The TGA-DTA spectra are shown in Figure 5. Thermogravimetric analysis (TGA) of selenium nanoparticles reveals an initial slight mass loss below 200 °C (6.7%), primarily attributed to the elimination of physically adsorbed water and residual volatile compounds. Significant degradation is observed in the 250 °C–320 °C range, reflecting the progressive decomposition of biomolecules from the plant extract (proteins, polyphenols, and other secondary metabolites) that act as stabilizers and capping agents around the SeNPs. The corresponding mass loss is approximately 36%. The third mass loss stage (320 °C–500 °C) can be attributed to the degradation of carbon residues and the oxidation of selenium-to-selenium dioxide (SeO_2_), which is volatile and can sublime at these temperatures. The final residue (∼23%) mainly corresponds to the stable mineral salts of the extract. In parallel, the DTA curve shows an endothermic effect below 147 °C, corresponding to the desorption of water, followed by exothermic peaks between 250 °C and 500 °C (Maximum temperatures at 338 °C and 450 °C), characteristic of the combustion or oxidation of organic constituents. All of these results confirm the good thermal stability of selenium nanoparticles, with the formation of a stable inorganic core after elimination of the organic constituents.

### 3.5 Effects of SeNPs on the ulcer index and ulceration protection of ethanol-induced gastric ulcers in rats

**FIGURE 6:**
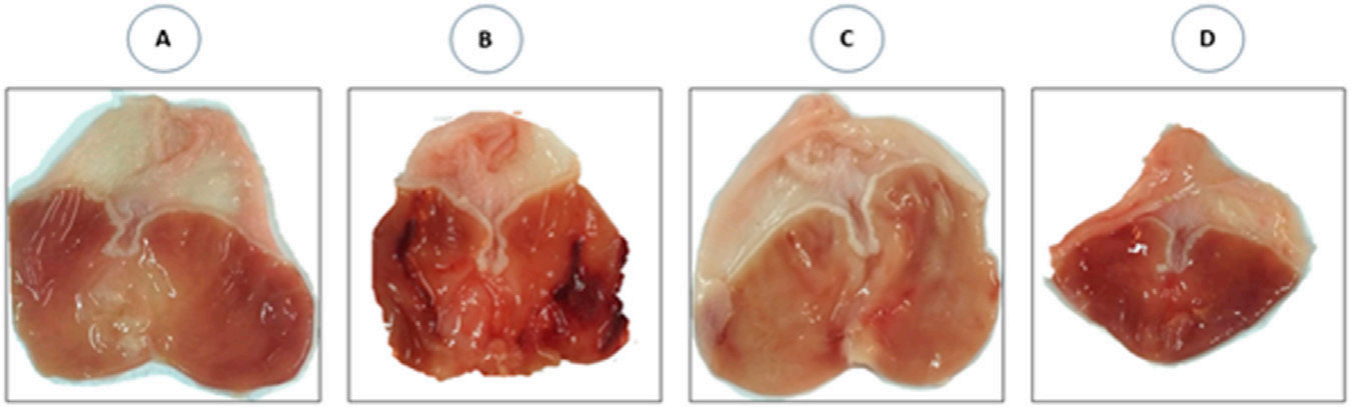
Photomacrographs view illustrating the effect of Se-NPs on the stomach tissue. **(A)** Control group showing gastric mucosa without signs of lesions, **(B)** Ulcer group (Ethanol 5 ml/Kg B.W) showing damaged gastric mucosa, with bloody ulcer streaks which were observed upon opening the stomach, **(C)** Se-NPs pretreated group (0.5 mg/kg B.W) showing mild gastric ulceration in ethanol-treated rats, **(D)** Se-NPs pretreated group (0.5 mg/kg BW) displaying a healthy gastric mucosa with no detectable injuries, lesions, or signs of edema.

The photomacrographs (Figure 6) view illustrating the effect of SeNPs on the stomach tissue were summarized following ethanol-induced gastric ulcer in rats. The control group exhibited a normal shape and color of their stomach, and there were no signs of ulceration (Figure 6A). However, the ethanol-exposed rats showed a damaged gastric mucosa, with bloody ulcer streaks which were observed upon opening the stomach (Figure 6B). Interestingly, when rats pretreated with SeNPs for seven consecutive days, their stomach tissue exhibited significant improvement. This was evidenced by a marked reduction in the gastric lesion index in ethanol-treated rats, as compared to those receiving only ethanol (Figure 6C). The stomach of rats from control positive group pretreated with SeNPs remained normal without any observed lesion, or edema (Figure 6D).

**FIGURE 7:**
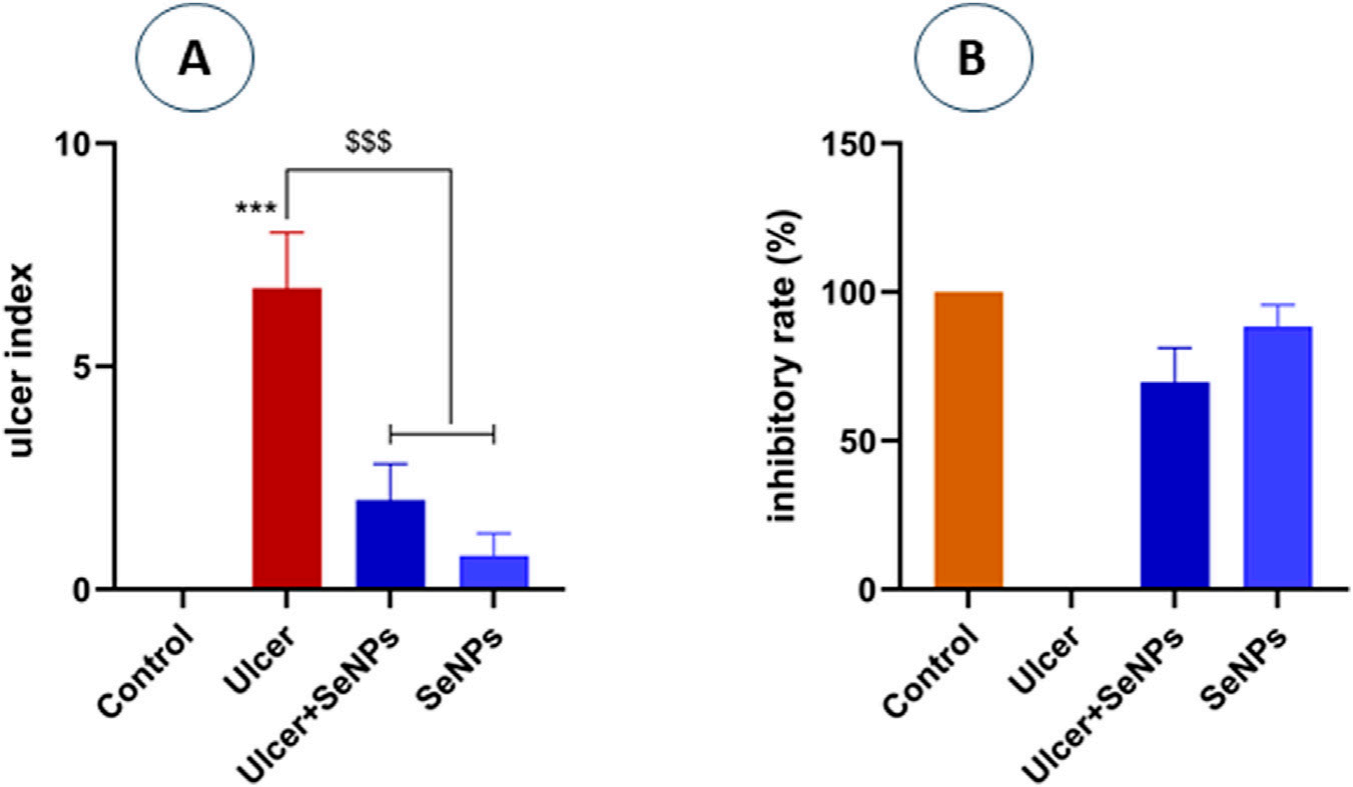
Panel **(A)** represents the ulcer index of the different groups. Panel **(B)** represents the inhibitory rate of SeNPs pretreatment. Control group (saline solution) without gastric ulcer; Ulcer group, induced by intragastric administration of ethanol; ulcer pretreated group with Se-NPs at a dose of 0.5 mg/kg B.W; SeNPs represent the positive group which rats were treated with Se-NPs (0.5 mg/kg B.W). Data are presented as Mean ± SD, n = 6. *** represents p < 0.001: significantly different from control group; ^$$$^ represents p < 0.001: significantly different from ulcer group.

As illustrated in Figure 7A, the ulcer index in ethanol-group rats was significantly (*p* < 0.001) higher when compared to the controls. In contrast, our results indicate that pretreatment of SeNPs at a dose of 0.5 mg/kg/*p.o* markedly (*p* < 0.001) reduced stomach lesions when compared to ethanol-treated rats (ulcer-group). The preventing ulceration effect of SeNPs was further assessed by the inhibitory rate of ulcer induction (Figure 7B), where SeNPs pretreatment significantly increased the protection rate reaching 69.64%, compared to the ethanol-treated rats.

### 3.6 SeNPs attenuates ethanol-induced gastric oxidative stress in rats

#### 3.6.1 Effects of SeNPs on the level of MDA gastric tissue

**FIGURE 8:**
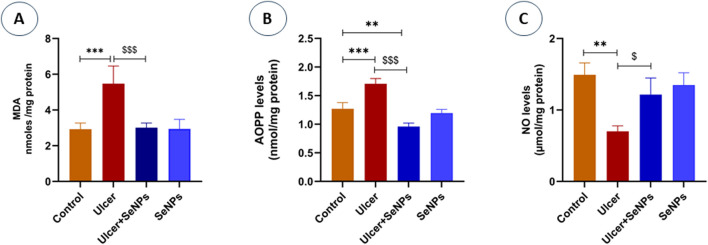
Effects of pre-treatment with SeNPs on MDA **(A)**, AOPP **(B)**, and NO **(C)** levels on the gastric tissue. Data are presented as Mean ± SD, n = 6. **, *** represents p < 0.01 and p < 0.001, respectively: significantly different from control group; ^$^, ^$$$^ represents p < 0.05 and p < 0.001, respectively: significantly different from ulcer group.

As indicated in Figure 8A, our results revealed a significant (*p* = 0.001) increase in lipid peroxidation (+69%) levels in the stomach homogenate of exposed rats to ethanol when compared to the controls. However, pretreatment of rats with SeNPs (0.5 mg/kg) restored MDA to near control values (*p* < 0.001). Administration of SeNPs (0.5 mg/kg) had no impact on MDA levels when compared to the controls.

#### 3.6.2 Estimation of protein oxidation

The effect of SeNPs on protein oxidation was shown in Figure 8B. Our findings reveal that ethanol significantly (*p* < 0.0001) increases protein oxidation, evidenced by a 34% elevation in advanced oxidation protein product (AOPP) levels in gastric tissue when compared to the controls. However, intragastric pretreatment of animals with SeNPs (0.5 mg/kg) significantly mitigated AOPP levels as compared to corresponding ethanol alone treated group.

#### 3.6.3 Effects of SeNPs on the level of NO gastric tissue

As shown in Figure 8C, intragastric ethanol administration to adult rats significantly (*p* = 0.0015) enhanced nitric oxide (NO) levels of gastric tissue, in comparison with the controls. On the other hand, pretreatment of rats with SeNPs (0.5 mg/kg), was able to reduce NO levels by 54.9% when compared to ethanol-induced gastric ulcer group. Administration of SeNPs alone did not influence NO levels when compared to the controls.

### 3.7 Effects of SeNPs on non-enzymatic antioxidant status

**FIGURE 9:**
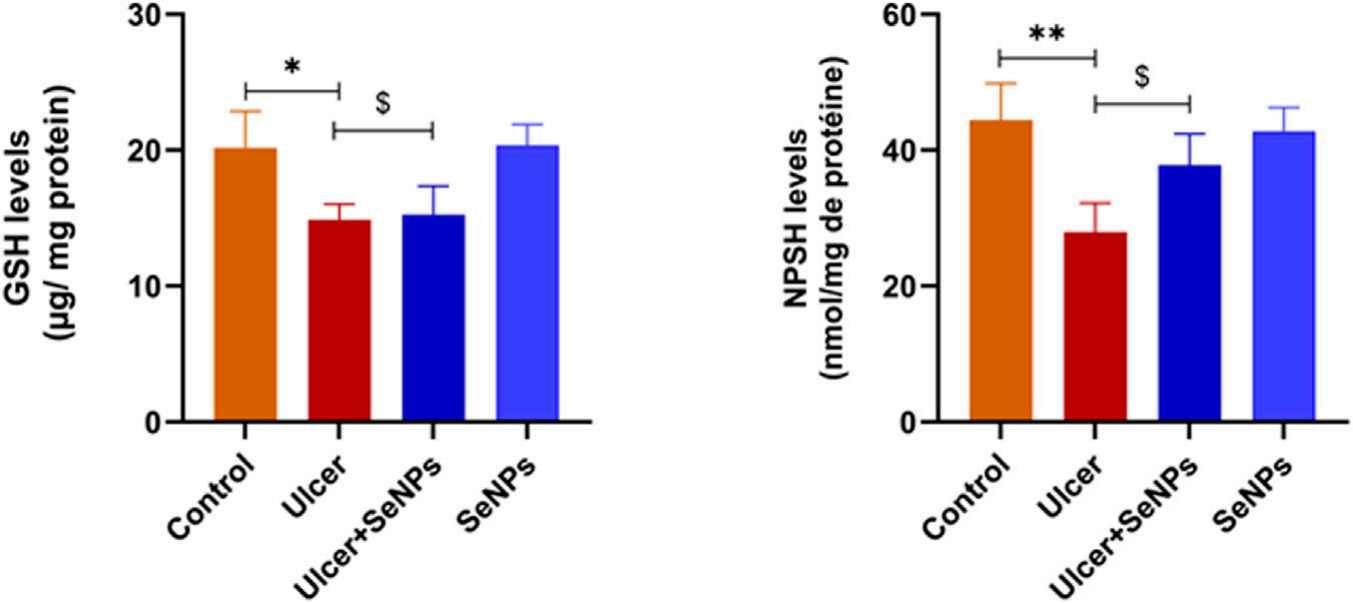
Effects of pre-treatment with SeNPs on reduced glutathione (GSH) and nonprotein thiols (NPSH) levels on the gastric tissue. Data are presented as Mean ± SD, n = 6. *, ** represents p < 0.05 and p < 0.01, respectively: significantly different from control group; ^$^ represents p < 0.05: significantly different from ulcer group.

As shown in Figure 9, in the ethanol-group, the gastric tissue levels of GSH and NPSH elevated considerably by 26% and 37% (*p* =0.0019 and *p* =0.0009) respectively, when distinguished to the control group. An incomplete improvement of GSH levels, without reaching control and SeNPs group’s values, was observed in the pretreated group when compared to the ethanol-group. Compared to the controls, intragastric SeNPs administration alone had no effect on the nonenzymatic antioxidant status.

### 3.8 Effects of SeNPs on enzymatic antioxidant status

**FIGURE 10:**
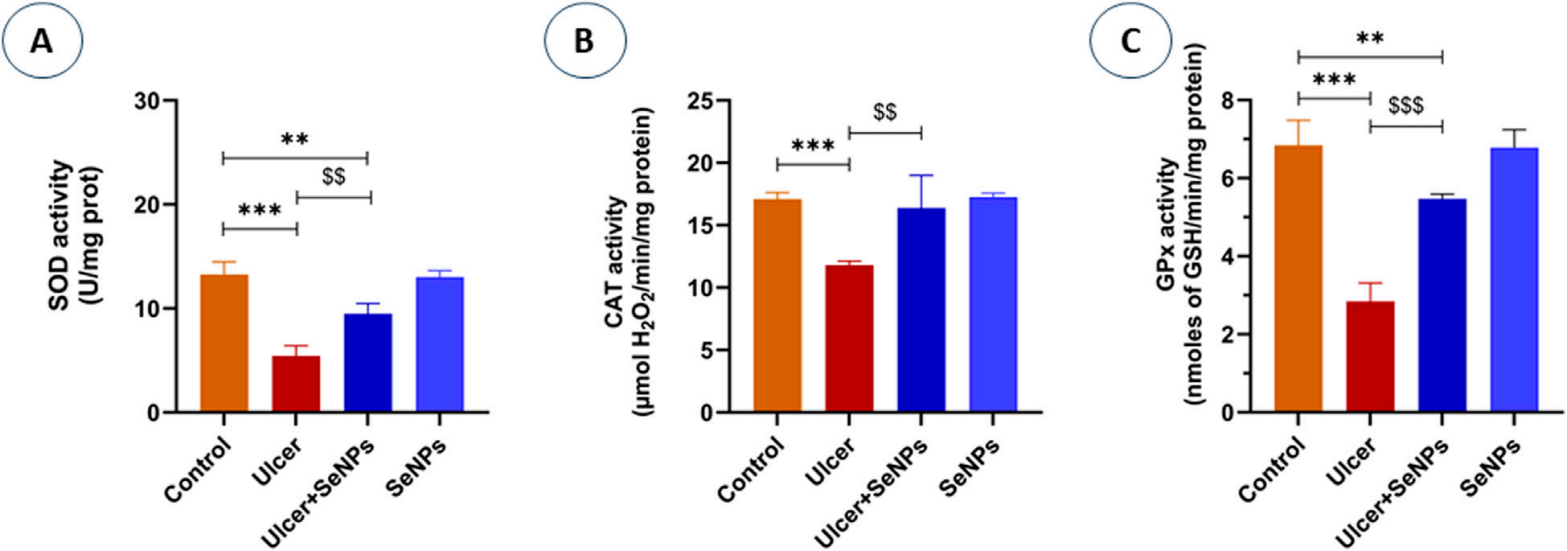
Effects of pre-treatment with SeNPs on Superoxide dismutase (SOD) (A), Catalase (CAT) (B), and Glutathione peroxidase (GPx) (C) activities on the gastric tissue. Data are presented as Mean ± SD, n = 6. **, *** represents p < 0.01 and p < 0.001, respectively: significantly different from control group; ^$$^, ^$$$^ represents p < 0.01 and p < 0.001, respectively: significantly different from ulcer group.

As mentioned in Figure 10, exposed adult rats to ethanol resulted in a significant decrease in the activity of SOD (Figure 10A) as well as CAT (Figure 10B) and GPx (Figure 10C) (−64, −40% and −58%) (*p* < 0.0001, *p* =0.0002 and *p* < 0.001) respectively, in stomach tissue when compared to the control group. However, pretreatment with SeNPs at doses of 0.5 mg/kg notably improved the activities of antioxidant enzymes which reached control values (*p* < 0.01). Furthermore, the enzymatic antioxidant status of the pretreated rats with SeNPs only at this dose remained unaffected.

### 3.9 Effects of SeNPs on hematological markers and CRP levels

The effects of pretreatment with SeNPs on hematological parameters are depicted in Table 1. Compared to the controls, the RBCs number, Hb concentration, Ht value and Plt count decreased significantly in ethanol-treated rats by 64, 8, 22% and 47% (*p* < 0.0001, *p* =0.0041, *p* < 0.0001 and *p* < 0.0001) respectively. No significant changes were recorded for MCV, MCH and MCHC, although there was a significant (*p* < 0.001) increase was recorded in WBC count by 115% in ethanol-treated rats, compared to the controls. Compared to the ulcer-group, those that ingested SeNPs show their hematological markers attenuated.

**TABLE 1:** Effect of pretreatment with SeNPs on hematological parameters in adult rats following gastric ulcer induction.

Parameters and groups	Controls	Ulcer	Ulcer + SeNPs	SeNPs
RBC (10^6^/µL)	8.728 ± 0.33	3.076 ± 0.515***	6.475 ± 0.8***$$$	8.691 ± 0.225
WBC (10^3^/μL)	6.826 ± 0.437	14.704 ± 0.221***	9.025 ± 0.845***$$$	6.774 ± 0.278
Hb (g/dL)	13.28 ± 0.571	12.2 ± 0.39**	13.07 ± 0.435$$	13.16 ± 0.248
Ht (%)	41.7 ± 1.44	32.36 ± 2.082***	38.21 ± 3.143*$	41.44 ± 0.487
MCV (μL/RBC)	47.48 ± 2.031	45.64 ± 3.260*	45.86 ± 1.768$	48.08 ± 2.409
MCH (pg/RBC)	16.92 ± 0.622	15.858 ± 0.540	16.048 ± 0.728*	16.81 ± 0.844
MCHC (g/dL)	38.36 ± 2.00	33.56 ± 2.518**	36.84 ± 2.426$	38.7 ± 2.804
Plt (10^3^/μL)	1,045.5 ± 55.979	546 ± 40.589***	869.2 ± 69.804**$$$	1,055.2 ± 106.805

Significant differences: values are means ± SD for 6 rats in each group.

*p < 0.05; **: p < 0.01; ***: p < 0.001 compared to Controls.

$: p < 0.05; $$: p < 0.01, $$$: p < 0.001 compared to Ulcer group.

**FIGURE 11:**
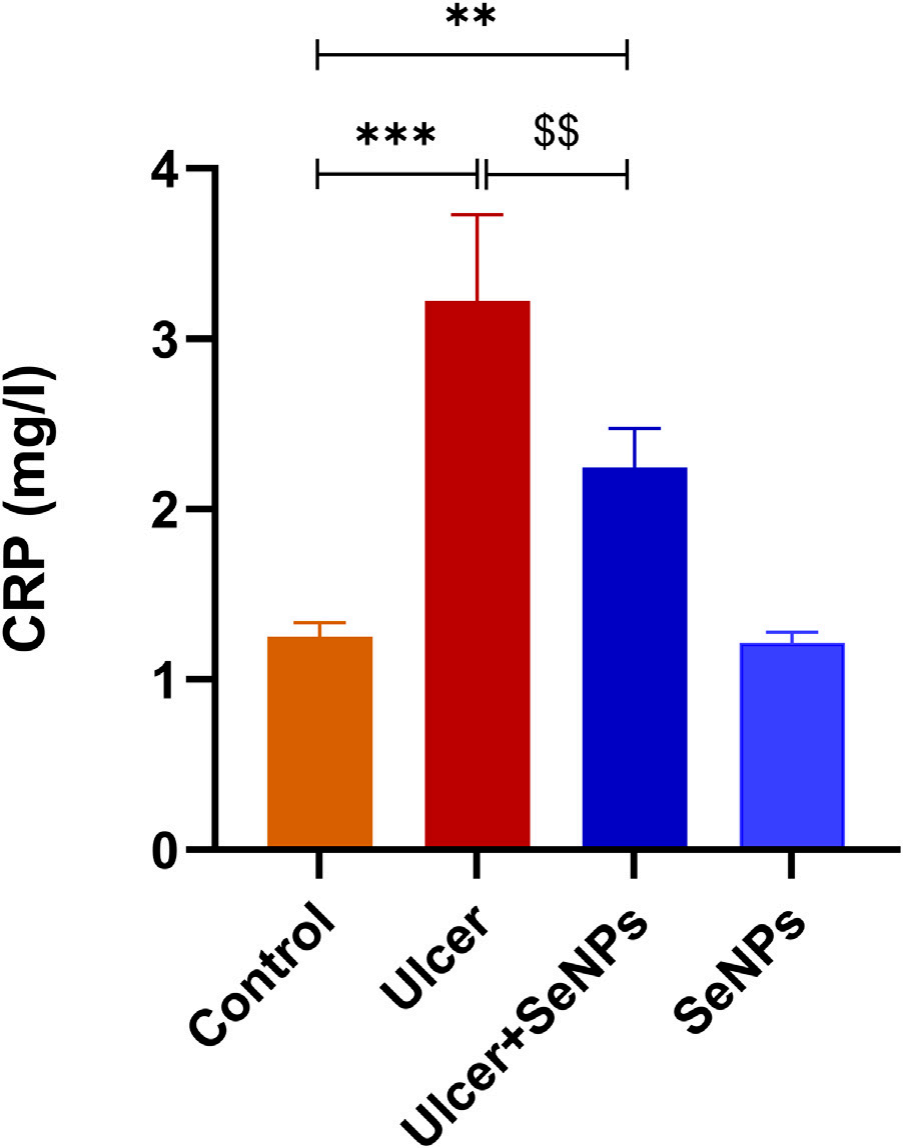
Effects of pre-treatment with Se-NPs on C Reactive Protein (CRP) Level on the gastric tissue. Data are presented as Mean ± SD, n = 6. ** represents p < 0.01 and p < 0.001, respectively: significantly different from control group; ^$$^ represents p < 0.01: significantly different from ulcer group.

On the other hand, as illustrated in Figure 11, plasma CRP levels in ethanol-treated rats increased markedly (*p* < 0.001), compared to the control group. However, the administration with SeNPs during seven consecutive days significantly (*p* < 0.01) improved plasma CRP levels in ulcer-group.

### 3.10 Histopathological findings

**FIGURE 12:**
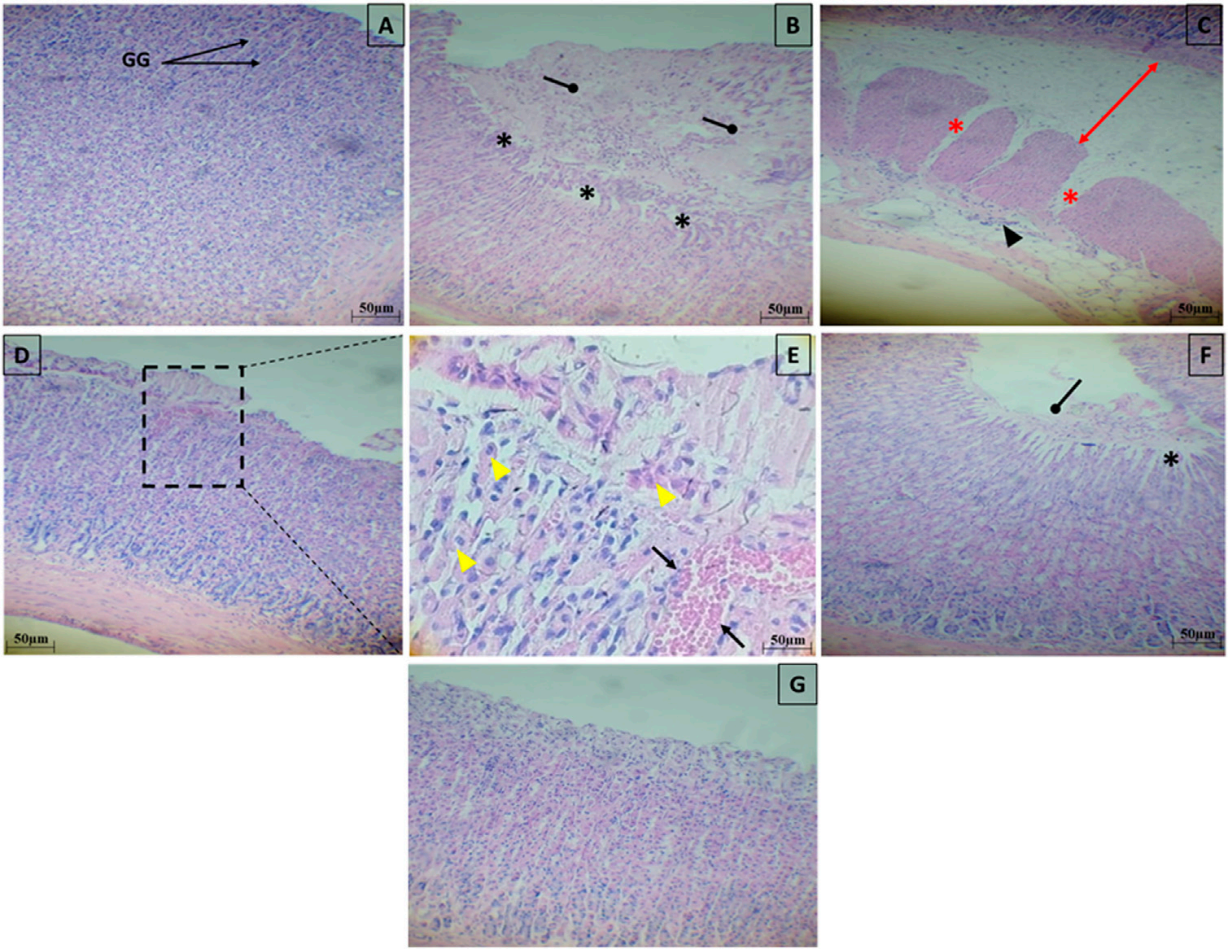
Representative photomicrograph of gastric tissue sections stained with H&E in ethanol-induced gastric ulcer in adult rats. Control group showed a normal architecture of the gastric tissue, with arranged glands and no inflammatory cell infiltration **(A)**. The gastric tissues of the ethanol-treated rats showed lesions on the surface of the epithelium (black star) with a lot of secretions of gastric mucus **(B)**, an extensive edema of submucosa (Red arrow), inflammatory infiltrate (ahead arrow) and a marked degeneration of muscular wall (Red star) **(C)**, hemorrhage in the superficial mucosal layer of stomach (Black arrow) and necrosis (yellow ahead arrow) **(D,E)**. Pretreatment with SeNPs at a dose of 0.5 mg/kg showed less mucosal disruption of the gastric tissue and epithelium lining **(F)**. Pretreatment with SeNPs only (0.5 mg/ kg) conserved the normal architecture of the gastric tissue **(G)** group.

Light microscopic analysis of the stomach in control group indicated a normal architecture of the gastric tissue, with arranged glands and no inflammatory cell infiltration (Figure 12A). However, several histopathological disorders have shown stomach tissue of rats exposed to ethanol with lesions on the surface of the epithelium (black star) with a lot of secretions of gastric mucus (Figure 12B). In addition, there was an extensive edema of submucosa (Red arrow), inflammatory infiltrate (ahead arrow) and a marked degeneration of muscular wall (Red star) (Figure 12C). Ethanol-treated group had also manifested hemorrhage in the superficial mucosal layer of stomach (Black arrow) and necrosis (yellow ahead arrow) (Figures 12D,E). Pretreatment with SeNPs at a dose of 0.5 mg/kg resulted in reduced mucosal injury the gastric tissue and epithelium lining (Figure 12F) compared to the ulcer group. Pretreatment with SeNPs only (0.5 mg/kg) conserved the normal histological structure of the gastric tissue (Figure 12G). These findings were corroborated by the histological scoring (Table 2) where the ethanol-treated group exhibited severe histological damage when compared to the control group. The administration of adult rats with SeNPs (0.5 mg/kg) reduced pathological changes from mild to moderate compared to ethanol-induced gastric ulcer.

**TABLE 2:** Scoring of the severity histopathological findings.

Histopathological changes/Groups	Controls	Ulcer	Ulcer + SeNPs	SeNPs
Lesions on the surface of the epithelium	–	+++	+	–
Hemorrhages	–	++	+	–
Inflammatory cell infiltration	–	++	-	–
Degeneration of muscular wall	–	+++	-	–

## 4 Discussion

Gastric ulcers and other gastrointestinal conditions are prevalent serious health concerns worldwide due to their elevated morbidity and mortality rates as well as a notable rise in their incidence over the past decades. Gastric ulcers were often managed through a broad-spectrum of conventional chemical-based therapies (Arfeen et al., 2024). However, the continued use of synthetic therapeutic medications is frequently associated with undesirable effects, prompting a growing need for safer and more natural treatment alternatives. Thus, researchers have increasingly focused their efforts on applying plant-based remedies based on nanotechnology for managing gastric ulcers, as these alternatives are considered relatively safe and free from adverse side effects (Prayoga et al., 2024). The present study was designed, for the first time, (i) to combine the extract of *Nigella sativa* L. with selenium in the nanoparticles, and (ii) to assess the possible effectiveness of SeNPs in adult rats subjected to ethanol-induced gastric ulceration. In this study, the successful biosynthesis of selenium nanoparticles (SeNPs) using *Nigella sativa* L. seed extract, as confirmed by the characteristic UV–Visible absorption peak around 230 nm, provides a strong foundation for evaluating their biological effects. The nanoscale dimensions and stable colloidal nature of these SeNPs, indicated by their surface plasmon resonance, suggest enhanced surface reactivity and bioavailability. Given their well-documented antioxidant and anti-inflammatory properties, these green-synthesized SeNPs are promising candidates for *in vivo* investigations to explore their protective potential against oxidative stress and tissue damage (Fan et al., 2020). In our study, intragastric administration of ethanol, as a necrotizing mediator was used to induce gastric ulcer model. It has been widely employed to assess the activity anti-ulcerative of various substances such as plants and drugs. The harmful effects of ethanol on the stomach were evident, leading to gastric mucosa barrier damage, inflammation, edema, hemorrhage, and ulceration. Such alterations subsequently disrupt the balance between mucosal protective mechanisms and overproduction of free radicals.

Our study demonstrates that single ethanol administration resulted in significant gastric mucosal injury, as shown by macroscopic examination and ulcer index determination in the gastric mucosal layer. The latter is considered the first line of defense against external xenobiotics (Lyon et al., 2024). As expected, in the ethanol-treated group, stomach pathological findings, stained with hematoxylin–eosin, showed gastrointestinal injury such as inflammatory cell infiltration, injury of the surface epithelial, hemorrhage, and erosion of gastric mucosa which are likely consequences of the heightened creation of ROS levels (Qiao et al., 2024; Liu et al., 2025). The SeNPs pretreatment at doses of 0.5 mg/kg BW decreased the development of gastric mucosal injuries, eventually demonstrated by a decrease in ulcer index, and higher inhibitory and therefore mitigate histological abnormalities following ethanol administration.

Such gastric mucosal impairments are associated with alterations in hematological parameters, which serve as crucial indicators of overall health in both animals and humans (Wu et al., 2023; Obeagu et al., 2024). Our results regarding blood analysis revealed substantial alterations in the stomach following gastric ulcer induction as indicated by a significant decrease in the RBC count, Ht, Hb levels, MCV and MCHC concentrations in the ethanol-treated group suggesting the occurrence of anemia. This may be attributed to several factors such as iron bioavailability, as the majority of this metal is associated with hemoglobin (Srole and Ganz, 2021). Reduced iron levels limit heme synthesis, which in turn inhibits erythropoiesis and decreases RBC production in the bone marrow, ultimately leading to anemia (Mueller and Scheller, 2023). Our findings were in line with previous studies showing that acetic acid and ibuprofen exposure induced the impairment of hematological parameters in rats (Saad and Qutob, 2023; Aliouche et al., 2024). On the other hand, our results revealed a significant increase in WBC following ethanol ingestion, while platelet counts (Plt) were markedly reduced, suggesting direct activation of immune response and the occurrence of secondary thrombocytosis (Han et al., 2023). Interestingly, we showed that pretreatment with SeNPs of ethanol-treated rats effectively prevented ulceration as assessed by a significant improvement of hematological parameters. Our results are supported by previous studies where SeNPs can mitigate diabetes in mice and protect against gastrointestinal damage induced by patulin in adult rats (Qiu et al., 2022; Karas et al., 2024).

C-reactive protein (CRP) is another well-known plasma indicator protein involved in inflammatory reactions. Upon xenobiotics exposure, CRP released by hepatocyte cells into the bloodstream in response to pro-inflammatory cytokines (Geyer et al., 2021). Herein, our findings showed a high level of plasma CRP in the ethanol-treated group when compared to controls, reflecting the presence of an intense inflammatory response in the site of ulceration. Our results were consistent with previous findings following a high dose of ibuprofen and indomethacin administration resulting in gastric ulcer and the development of epithelium inflammation, which ultimately leads to a rising in WBC levels (Lisna and Stepchenko, 2022; Uslu et al., 2022). Importantly, we have shown that SeNPs pretreatment reduces CRP thereby promoting ulcer healing. This protective effect is attributed in part to the bioactive constituents of *Nigella sativa* L. particularly the thymoquinone, which exhibited antimicrobial activity against *H. pylori* and, consequently, anti-ulcer properties (Cherrada et al., 2024).

Gastric inflammatory response, including CRP elevation, is involved with the formation of reactive oxygen species (ROS), which can ultimately lead to disruption of oxidant/antioxidant balance (Guo et al., 2024). It is well known that free radicals can react with lipids to form lipid peroxides (Zheng et al., 2024), which in turn disrupt membrane permeability and ultimately resulting in cell death and or apoptosis (An et al., 2024). In our present study, single-dose ethanol administration to adult rats enhanced free radical production, as evidenced by the raised lipid peroxidation (MDA) indicating cellular damage and increased in protein oxidation biomarker (AOPP) (Ortega-Pérez et al., 2024). Excessive production of cellular ROS not only damages lipids and proteins but also compromises the body’s endogenous antioxidant defense system (Mondal and Bandyopadhyay, 2024). Both enzymatic and non-enzymatic antioxidant systems work together to enhance gastric protection and promote healing effects (Beiranvand et al., 2021). In the present study, the system’s imbalance was evident through a reduction in non-enzymatic antioxidants, in particular GSH and NPSH. Cysteine-rich polypeptides and tripeptides of low molecular weight are abundant in the gastric mucosa where they displayed a vital role in preserving mucosal layer integrity (Araújo et al., 2024). Further, in our current study, ethanol administration also compromised the activities of key antioxidant enzymes including SOD, CAT, and GPx. These results support earlier findings that ethanol exposure can reduce the gastric mucosa’s defenses and increases lipid peroxide formation, resulting in a notable decline in the activity of key enzymes such as SOD, CAT and GPx in gastric tissue (Bahramikia and Izadi, 2023). Ethanol is well known to directly induce damages in the gastric mucosal barrier, increases its permeability, and alters gastric acid cells, resulting in stomach injuries. Pretreatment with SeNPs significantly enhanced gastric ulcers through enzymatic antioxidant activities improvement, suggesting its protective effect against oxidative stress. As previously reported, *Nigella sativa* L. seed aqueous extract enhances gastric ulcer healing by boosting antioxidant enzymes (SOD, CAT, GPx), restoring gastric mucus and NP-SH levels, and protecting the mucosa from oxidative damage. Its anti-ulcer activity is mainly due to bioactive compounds like thymoquinone, which inhibits acid secretion, increases mucus, and combats *H. pylori*, while thymohydroquinone and thymol contribute antioxidant and anti-inflammatory effects (Al Mofleh et al., 2008; Khan et al., 2016; Paseban et al., 2019). However, its clinical use is limited due to its poor bioavailability. Selenium nanoparticles synthesized with *Nigella sativa* L. improve bioavailability and antioxidant activity, enabling targeted delivery and enhanced gastric protection, making them a more effective therapeutic option (Sowunmi et al., 2025). SeNPs play a crucial role in the antioxidant defense system by directly scavenging reactive oxygen species (ROS) and enhancing the activity of selenoenzymes such as glutathione peroxidase (GPx) and thioredoxin reductase, thereby reducing oxidative stress (Azmoonfar et al., 2024), alleviating liver injury, and lowering enzyme levels related to liver function in rats subjected to gamma-ray radiation (Sohrabi et al., 2020). Additionally, SeNPs improve cellular redox balance by activating antioxidant pathways and inhibiting oxidative damage, making them effective in protecting cells against oxidative stress-related injury (Chen et al., 2023; Nogales et al., 2024).

Nitric oxide (NO), among important mucosal protective factors and act as a vasodilator. Upon toxic substance exposure, the blood flow increases drastically in response to hyperaemia leading to a high vulnerability of the gastric mucosa. NO is also implicated in stimulation of gastric mucus, maintenance of gastric blood flow, bicarbonate secretion, and inhibition of inflammatory process. In the present study, the reduction of NO in ethanol-treated rats may result from oxidative damage. This reduction can lead to compromised blood flow and increased risk of ulceration. Our findings align with previous studies who have reported that ethanol administration decreases NO levels in gastric tissue, highlighting the complex role of NO in gastric health and disease (Moawad et al., 2019; AbdelAziz et al., 2021; Liu et al., 2021). Importantly, our present work reported that SeNPs pretreatment could prevent oxidative damage in gastric tissue and accelerate the process of ulcer healing. Previous study have reported that SeNPs may alleviate the kidney damage induced by vancomycin and improve the level of nitric oxide (Mehanna et al., 2022).

## 5 Conclusion

Our findings summarized for the first time that pretreatment of SeNPs successfully alleviate ethanol-induced gastric ulcer reduced mucosal damage, oxidative stress, and performed the redox imbalance and histopathological alterations. These positive outcomes are probably due to its antioxidative (through improvement of the endogenous antioxidant system), anti-inflammatory activities (by normalization of the level of WBC and NO). Moreover, the hematological parameters of ethanol-induced gastric ulcer rats underwent marked improvement after pretreatment with SeNPs. Although the promising outcomes, further research are needed to examine genes related to the antioxidant and anti-inflammation genes in order to clarify the underlying protective effect of SeNPs to mitigate gastric ulcers. Additionally, their long-term safety, bioavailability, and their potential synergistic effects with other therapeutic agents are essential. Our findings may represent a potential therapeutic approach to further developed nanoparticles-based plants as an effective alternative or complementary to conventional medication in the prevention of gastric ulcers.

## Data Availability

The raw data supporting the conclusions of this article will be made available by the authors, without undue reservation.

## Abbreviations

XRD, X-ray diffraction analysis; SEM, scanning electron microscope; FT-IR, Fourier transform infrared; TGA-DTA, Thermal-gravimetric analysis; NO, nitric oxide; NSAIDs, nonsteroidal anti-inflammatory drugs; SeNPs, Selenium nanoparticles; Na_2_SeO_3_, Sodium selenite.

## References

[B1] AbdelAziz E. Y. Tadros M. G. Menze E. T. (2021). The effect of metformin on indomethacin-induced gastric ulcer: involvement of nitric oxide/Rho kinase pathway. Eur. J. Pharmacol. 892, 173812. 10.1016/j.ejphar.2020.173812 33345855

[B2] Ahmad A. Javad S. Iqbal S. Shahzadi K. Gatasheh M. K. Javed T. (2024). Alleviation potential of green-synthesized selenium nanoparticles for cadmium stress in Solanum lycopersicum L: modulation of secondary metabolites and physiochemical attributes. Plant Cell Rep. 43, 113. 10.1007/s00299-024-03197-9 38573519

[B3] Ahmed M. E. Alzahrani K. K. Fahmy N. M. Almutairi H. H. Almansour Z. H. Alam M. W. (2025). Colistin-conjugated selenium nanoparticles: a dual-action strategy against drug-resistant infections and cancer. Pharmaceutics 17, 556. 10.3390/pharmaceutics17050556 40430850 PMC12114847

[B4] Al Mofleh I. A. Alhaider A. A. Mossa J. S. Al-Sohaibani M. O. Al-Yahya M. A. Rafatullah S. (2008). Gastroprotective effect of an aqueous suspension of Black cumin Nigella sativa on necrotizing agents-induced gastric injury in experimental animals. Saudi J. Gastroenterol. 14, 128–134. 10.4103/1319-3767.41731 19568521 PMC2702910

[B5] Aliouche N. Sifour M. Kebsa W. Khennouf T. Ercan F. Ouled-Haddar H. (2024). Prophylactic effect and antiulcerogenic potential of probiotic Lactiplantibacillus plantarum E1K2R2 and its exopolysaccharide against ibuprofen-induced acute gastric ulcer. Probiotics Antimicrob. Proteins. 10.1007/s12602-024-10321-4 39008160

[B6] Alvi G. B. Iqbal M. S. Ghaith M. M. S. Haseeb A. Ahmed B. Qadir M. I. (2021). Biogenic selenium nanoparticles (SeNPs) from citrus fruit have anti-bacterial activities. Sci. Rep. 11, 4811. 10.1038/s41598-021-84099-8 33637796 PMC7910555

[B7] Ambreen Khan M. A. Raza A. Yousaf R. Ali H. Darwish H. (2024). Impact of zinc oxide nanoparticles on biosynthesis of thymoquinone in cell cultures of *Nigella sativa* . Plant Nano Biol. 10, 100109. 10.1016/j.plana.2024.100109

[B8] An X. Yu W. Liu J. Tang D. Yang L. Chen X. (2024). Oxidative cell death in cancer: mechanisms and therapeutic opportunities. Cell Death Dis. 15, 556–20. 10.1038/s41419-024-06939-5 39090114 PMC11294602

[B9] Araújo R. P. N. da Silva Freitas F. V. Nunes D. B. da Silva Brito A. K. da Costa D. S. de Sousa D. P. (2024). Investigating the pharmacological potential of phytol on experimental models of gastric ulcer in rats. Schmiedeb. Arch. Pharmacol. 397, 7757–7766. 10.1007/s00210-024-03085-9 38717706

[B10] Arfeen M. Srivastava A. Srivastava N. Khan R. A. Almahmoud S. A. Mohammed H. A. (2024). Design, classification, and adverse effects of NSAIDs: a review on recent advancements. Bioorg. Med. Chem. 112, 117899. 10.1016/j.bmc.2024.117899 39217686

[B11] Azmoonfar R. Moslehi M. Shahbazi-Gahrouei D. (2024). Radioprotective effect of selenium nanoparticles: a mini review. IET Nanobiotechnol 2024, 5538107. 10.1049/2024/5538107 38863968 PMC11095073

[B12] Bahramikia S. Izadi R. (2023). Plant-based green synthesis of nanoparticles as an effective and safe treatment for gastric ulcer. Inflammopharmacol 31, 2843–2855. 10.1007/s10787-023-01367-x 37921959

[B13] Beiranvand M. Bahramikia S. (2020). Ameliorating and protective effects *mesalazine* on ethanol-induced gastric ulcers in experimental rats. Eur. J. Pharmacol. 888, 173573. 10.1016/j.ejphar.2020.173573 32956646

[B14] Beiranvand M. Bahramikia S. Dezfoulian O. (2021). Evaluation of antioxidant and anti-ulcerogenic effects of Eremurus persicus (Jaub & Spach) boiss leaf hydroalcoholic extract on ethanol-induced gastric ulcer in rats. Inflammopharmacol 29, 1503–1518. 10.1007/s10787-021-00868-x 34435283

[B15] Ben Amara F. Jemli S. Marques H. C. Akermi S. Ennouri M. Smaoui S. (2025). Preparation and characterization of inclusion complexes of Nigella sativa seed oil with β-cyclodextrin: *in vitro* biological applications and *in silico* assessment. Biomass Conv. bioref. 15, 12287–12300. 10.1007/s13399-024-06069-0

[B16] Chen N. Yao P. Zhang W. Zhang Y. Xin N. Wei H. (2023). Selenium nanoparticles: enhanced nutrition and beyond. Crit. Rev. Food Sci. Nutr. 63, 12360–12371. 10.1080/10408398.2022.2101093 35848122

[B17] Cherrada N. Chemsa A. E. Gheraissa N. Laib I. Gueboudji Z. El-Shazly M. (2024). Gastroprotective efficacy of North African medicinal plants: a review on their therapeutic potential for peptic ulcers. Food Sci. Nutr. 12, 8793–8824. 10.1002/fsn3.4536 39619964 PMC11606823

[B18] Choi W.-T. Lauwers G. Y. Slavik T. (2024). Inflammatory disorders of the stomach. In: Morson and dawson’s gastrointestinal pathology. Hoboken, New Jersey: John Wiley & Sons, Ltd. p. 135–194. 10.1002/9781119423195.ch11

[B19] Daoudi H. Bouafia A. Laouini S. E. Meneceur S. Fellah M. Iqbal A. (2024). *In vitro* and *in silico* study of biosynthesized silver nanoparticles using *Nigella sativa* extract against SARS-CoV-2 and *Candida albicans* . J. Mol. Liq. 405, 125059. 10.1016/j.molliq.2024.125059

[B20] Dewangan H. K. Singh N. Kumar Megh S. Singh S. Lakshmi (2022). Optimisation and evaluation of Gymnema sylvestre extract loaded polymeric nanoparticles for enhancement of *in vivo* efficacy and reduction of toxicity. J. Microencapsul. 39, 125–135. 10.1080/02652048.2022.2051625 35282781

[B21] El Messaoudi N. Ciğeroğlu Z. Şenol Z. M. Kazan-Kaya E. S. Fernine Y. Gubernat S. (2025). Green synthesis of CuFe2O4 nanoparticles from bioresource extracts and their applications in different areas: a review. Biomass Conv. Bioref. 15, 99–120. 10.1007/s13399-023-05264-9

[B22] El-Batal A. I. Ragab Y. M. Amin M. A. El-Roubi G. M. Mosallam F. M. (2021). Investigating the antimicrobial, antioxidant and cytotoxic activities of the biological synthesized glutathione selenium nano-incorporation. Biometals 34, 815–829. 10.1007/s10534-021-00309-w 33895912

[B23] Fan D. Li L. Li Z. Zhang Y. Ma X. Wu L. (2020). Biosynthesis of selenium nanoparticles and their protective, antioxidative effects in streptozotocin induced diabetic rats. Sci. Technol. Adv. Mater. 21, 505–514. 10.1080/14686996.2020.1788907 32939175 PMC7476508

[B24] Geyer C. E. Newling M. Sritharan L. Griffith G. R. Chen H.-J. Baeten D. L. P. (2021). C-Reactive protein controls IL-23 production by human monocytes. Int. J. Mol. Sci. 22, 11638. 10.3390/ijms222111638 34769069 PMC8583945

[B25] Guo Q. Lu T. Zhang M. Wang Q. Zhao M. Wang T. (2024). Protective effect of berberine on acute gastric ulcer by promotion of tricarboxylic acid cycle-mediated arachidonic acid metabolism. J. Inflamm. Res. 17, 15–28. 10.2147/JIR.S436653 38193042 PMC10772049

[B26] Hadian M. Fathi M. Mohammadi A. Eskandari M. H. Asadsangabi M. Pouraghajan K. (2024). Characterization of chitosan/Persian gum nanoparticles for encapsulation of *Nigella sativa* extract as an antiviral agent against avian coronavirus. Int. J. Biol. Macromol. 265, 130749. 10.1016/j.ijbiomac.2024.130749 38467218

[B27] Han M. Lin W. Huang S. Lin Z. Li K. (2023). Association between plasma metal elements and platelet dysfunction in trauma-induced coagulopathy rat model. J. Trace Elem. Med. Biol. 79, 127210. 10.1016/j.jtemb.2023.127210 37229983

[B28] Hannan M. A. Zahan M. S. Sarker P. P. Moni A. Ha H. Uddin M. J. (2021). Protective effects of Black cumin (Nigella sativa) and its bioactive constituent, thymoquinone against kidney injury: an aspect on pharmacological insights. Int. J. Mol. Sci. 22, 9078. 10.3390/ijms22169078 34445781 PMC8396533

[B29] Islam Md. R. Akash S. Jony M. H. alam Md. N. Nowrin F. T. Rahman Md. M. (2023). Exploring the potential function of trace elements in human health: a therapeutic perspective. Mol. Cell Biochem. 478, 2141–2171. 10.1007/s11010-022-04638-3 36637616

[B30] Kamnev A. A. Dyatlova Y. A. Kenzhegulov O. A. Vladimirova A. A. Mamchenkova P. V. Tugarova A. V. (2021). Fourier transform infrared (FTIR) spectroscopic analyses of microbiological samples and biogenic selenium nanoparticles of microbial origin: sample preparation effects. Molecules 26, 1146. 10.3390/molecules26041146 33669948 PMC7924863

[B31] Karas R. A. Alexeree S. Elzohery N. Abdel-Hafez S. H. Attia Y. A. (2024). Antidiabetic potential of selenium nanoparticles and plasma-rich platelets in diabetic mice. Appl. Biol. Chem. 67, 62. 10.1186/s13765-024-00907-5

[B32] Khan S. A. Khan A. M. Karim S. Kamal M. A. Damanhouri G. A. Mirza Z. (2016). Panacea seed “Nigella”: a review focusing on regenerative effects for gastric ailments. Saudi J. Biol. Sci. 23, 542–553. 10.1016/j.sjbs.2014.10.001 27298589 PMC4890198

[B33] Khan N. Khushtar M. Rahman M. A. Kaish M. Ajmal M. (2024). Amelioration of gastric ulcer using a hydro-alcoholic extract of *Mangifera indica* in sprague dawley rats by prevention of muco-oxidative stress. Pharmacol. Res. - Mod. Chin. Med. 11, 100442. 10.1016/j.prmcm.2024.100442

[B34] Khitouni M. (2003). The effects of boron additions on the disordering and crystallite refinement of NI3AI powders during mechanical milling. Ann. de Chimie Sci. des Matériaux 28, 17–29. 10.1016/S0151-9107(03)00103-X

[B35] Lisna A. Y. Stepchenko L. M. (2022). Haematological changes in laboratory rats with Ibuprofen-induced gastric ulcer treated with humilid. Theor. Appl. Veterinary Med. 10, 15–22. 10.32819/2022.10008

[B36] Liu Y. Sui D. Fu W. Sun L. Li Y. Yu P. (2021). Protective effects of polysaccharides from Panax ginseng on acute gastric ulcers induced by ethanol in rats. Food Funct. 12, 2741–2749. 10.1039/D0FO02947E 33681872

[B37] Liu X. Ge J. Yang X. Chang Z. Ma X. Cheng S. (2025). Puerarin modulates the P62-Keap1-NRF2 pathway and enhances CA7 function to inhibit ferroptosis in ethanol-induced gastric mucosal injury. Food Biosci. 66, 106127. 10.1016/j.fbio.2025.106127

[B38] Lyon K. Sidar B. Strupulis C. Helm R. Davis B. Brown J. (2024). Human gastric organoid-derived mucus shares key properties with native gastric mucus and inhibits motility of Helicobacter pylori. Physiology 39, 2011. 10.1152/physiol.2024.39.S1.2011

[B39] Mehanna E. T. Khalaf S. S. Mesbah N. M. Abo-Elmatty D. M. Hafez M. M. (2022). Anti-oxidant, anti-apoptotic, and mitochondrial regulatory effects of selenium nanoparticles against vancomycin induced nephrotoxicity in experimental rats. Life Sci. 288, 120098. 10.1016/j.lfs.2021.120098 34715137

[B40] Mirzaei F. Abbasi E. Mirzaei A. Hosseini N. F. Naseri N. Khodadadi I. (2025). Toxicity and hepatoprotective effects of ZnO nanoparticles on normal and high-fat diet-fed rat livers: mechanism of action. Biol. Trace Elem. Res. 203, 199–217. 10.1007/s12011-024-04108-5 38441796

[B41] Moawad H. El Awdan S. A. Sallam N. A. El-Eraky W. I. Alkhawlani M. A. (2019). Gastroprotective effect of cilostazol against ethanol- and pylorus ligation–induced gastric lesions in rats. Schmiedeb. Arch. Pharmacol. 392, 1605–1616. 10.1007/s00210-019-01699-y 31372695

[B42] Mondal S. Bandyopadhyay A. (2024). Antioxidants in mitigating phthalate-induced male reproductive toxicity: a comprehensive review. Chemosphere 364, 143297. 10.1016/j.chemosphere.2024.143297 39245218

[B43] Mueller S. Scheller M. (2023). Ethanol-mediated bone marrow toxicity and impaired erythropoiesis: implications for alcohol-related liver disease. In: Mueller S. Heilig M. , editors. Alcohol and alcohol-related diseases. Cham: Springer International Publishing. p. 1107–1130. 10.1007/978-3-031-32483-3_58

[B44] Mumtaz F. Farag B. M. Farahat M. A. Farouk F. A. Aarif M. Y. Eltresy M. H. (2024). Leek (Allium ampeloprasum var. kurrat) aqueous extract loaded on selenium nanoparticles protects against testis and brain injury induced by mercuric chloride in rats. J. Sci. Food Agric. 104, 9062–9075. 10.1002/jsfa.13733 38993070

[B45] Nauroze T. Ali S. Kanwal L. Ara C. Akbar Mughal T. Andleeb S. (2023). Ameliorative effect of *Nigella sativa* conjugated silver nanoparticles against chromium-induced hepatotoxicity and renal toxicity in mice. Saudi J. Biol. Sci. 30, 103571. 10.1016/j.sjbs.2023.103571 36844642 PMC9944502

[B46] Nogales F. Pajuelo E. Romero-Herrera I. Carreras O. Merchán F. Carrasco López J. A. (2024). Uncovering the role of selenite and selenium nanoparticles (SeNPs) in adolescent rat adipose tissue beyond oxidative balance: transcriptomic analysis. Antioxidants 13, 750. 10.3390/antiox13060750 38929188 PMC11200624

[B47] Obeagu E. I. Igwe M. C. Obeagu G. U. (2024). Oxidative stress’s impact on red blood cells: unveiling implications for health and disease. Medicine 103, e37360. 10.1097/MD.0000000000037360 38428906 PMC10906601

[B48] Orosel D. Leynaud O. Balog P. Jansen M. (2004). Pressure–temperature phase diagram of SeO2. Characterization of new phases. J. Solid State Chem. 177, 1631–1638. 10.1016/j.jssc.2003.12.028

[B49] Ortega-Pérez L. G. Hernández-Soto J. A. Padilla-Avalos O. Ayala-Ruiz L. A. Magaña-Rodríguez O. R. Piñón-Simental J. S. (2024). Role of Callistemon citrinus leaf phytosomes against oxidative stress and inflammation in rats fed with a high-fat-fructose diet. Antioxidants 13, 1263. 10.3390/antiox13101263 39456515 PMC11504497

[B50] Paseban M. Niazmand S. Soukhtanloo M. Meybodi N. T. (2019). The preventive effect of Nigella sativa seed on gastric ulcer induced by indomethacin in rat. J. Herbmed Pharmacol. 9, 12–19. 10.15171/jhp.2020.02

[B51] Prayoga D. K. Aulifa D. L. Budiman A. Levita J. (2024). Plants with anti-ulcer activity and mechanism: a review of preclinical and clinical studies. Drug Des. Dev. Ther. 18, 193–213. 10.2147/DDDT.S446949 38318501 PMC10840521

[B52] Pyrzynska K. (2024). Plant extracts for production of functionalized selenium nanoparticles. Materials 17, 3748. 10.3390/ma17153748 39124412 PMC11313377

[B53] Pyrzynska K. Sentkowska A. (2024). Selenium species in diabetes mellitus type 2. Biol. Trace Elem. Res. 202, 2993–3004. 10.1007/s12011-023-03900-z 37880477 PMC11074226

[B54] Qiao K. Song Z. Liang L. Zhou X. Feng X. Xu Y. (2024). Exploring the underlying mechanisms of preventive treatment related to dietary factors for gastric diseases. J. Agric. Food Chem. 72, 17782–17801. 10.1021/acs.jafc.4c05361 39102359

[B55] Qiu Y. Chen X. Chen Z. Zeng X. Yue T. Yuan Y. (2022). Effects of selenium nanoparticles on preventing patulin-induced liver, kidney and gastrointestinal damage. Foods 11, 749. 10.3390/foods11050749 35267382 PMC8909330

[B56] Rua R. M. Nogales F. Carreras O. Ojeda M. L. (2023). Selenium, selenoproteins and cancer of the thyroid. J. Trace Elem. Med. Biol. 76, 127115. 10.1016/j.jtemb.2022.127115 36481604

[B57] Saad R. A. Qutob H. M. (2023). Alterations in hemostatic and hematological parameters after gastric ulcer induction in rats. Possible role of IL-6 and TNF-α. J. Evol. Biochem. Phys. 59, 82–93. 10.1134/S0022093023010076

[B58] Shaik R. A. (2024). Parthenolide alleviates indomethacin-induced gastric ulcer in rats *via* antioxidant, anti-inflammatory, and antiapoptotic activities. Schmiedeb. Arch. Pharmacol. 397, 7683–7695. 10.1007/s00210-024-03110-x 38703207

[B59] Sharifi-Rad M. Pohl P. Epifano F. Álvarez-Suarez J. M. (2020). Green synthesis of silver nanoparticles using Astragalus tribuloides delile. Root extract: characterization, antioxidant, antibacterial, and anti-inflammatory activities. Nanomaterials 10, 2383. 10.3390/nano10122383 33260441 PMC7760762

[B60] Sharifi-Rad M. Kishore Mohanta Y. Pohl P. Nayak D. Messaoudi M. (2024). Facile phytosynthesis of gold nanoparticles using *Nepeta bodeana* bunge: evaluation of its therapeutics and potential catalytic activities. J. Photochem. Photobiol. A Chem. 446, 115150. 10.1016/j.jphotochem.2023.115150

[B61] Shoman N. A. Salama A. Abbas F. G. Mourad H. H. Abbas H. A. (2025). LC-MS/MS profiling and immunomodulatory potential of green-synthesized zinc oxide nanoparticles using *Cordia sebestena* leaves against chromium-induced acute lung injury in rats. J. Drug Deliv. Sci. Technol. 106, 106750. 10.1016/j.jddst.2025.106750

[B62] Sohrabi A. Tehrani A. A. Asri-Rezaei S. Zeinali A. Norouzi M. (2020). Histopathological assessment of protective effects of selenium nanoparticles on rat hepatocytes exposed to gamma radiation. Vet. Res. Forum 11, 347–353. 10.30466/vrf.2018.93499.2260 33643587 PMC7904117

[B63] Sowunmi K. Abimbola O. M. Abah M. A. Sulaiman L. O. Izudike O. E. Ekele U. J. (2025). Green synthesis, photo-physical characterization, and evaluation of *in-vitro* antioxidant, anti-inflammatory, and antidiabetic properties of selenium nanoparticles derived from Nigella sativa seeds. JRANN 1, 1–07. 10.19080/JRANN.2025.01.555567

[B64] Srole D. N. Ganz T. (2021). Erythroferrone structure, function, and physiology: iron homeostasis and beyond. J. Cell. Physiology 236, 4888–4901. 10.1002/jcp.30247 33372284 PMC8026552

[B65] Stella P. C. R. Praba A. A. Nivetha A. (2025). Phytofabrication of selenium nanoparticles for investigating their efficacy as nanotherapeutics against lung carcinoma cells and bacterial pathogens. Orient. J. Chem. 41, 222–230. 10.13005/ojc/410127

[B66] Turkyilmaz I. B. Bayrak B. B. Sacan O. Mutlu O. Akev N. Yanardag R. (2021). Zinc supplementation restores altered biochemical parameters in stomach tissue of STZ diabetic rats. Biol. Trace Elem. Res. 199, 2259–2265. 10.1007/s12011-020-02352-z 32820429

[B67] Uslu H. Uslu G. A. Uslu H. Uslu G. A. (2022). Gastroprotective effects of Taraxacum officinale (Dandelion) extract indomethacin-induced gastric ulcer model. GSC Biol. Pharm. Sci. 20, 074–081. 10.30574/gscbps.2022.20.3.0347

[B68] Uthansingh K. Sidoine K. Patnaik S. K. Pati G. K. Padhy R. N. (2024). Natural product-based treatment for gastritis. In: Sharma T. Sahoo C. R. Bhattacharya D. Pati S. , editors. Natural products for antibacterial drug development: recent advancement of computational approach. Singapore: Springer Nature. p. 167–197. 10.1007/978-981-97-9634-2_8

[B69] Wang K. Fang Q. He P. Tu Y. Liu Z. Li B. (2024). Unveiling the potential of selenium-enriched tea: compositional profiles, physiological activities, and health benefits. Trends Food Sci. Technol. 145, 104356. 10.1016/j.tifs.2024.104356

[B70] Waseem S. Nisa Z. U. Zeeshan T. Ali M. D. Begum T. Kayani Z. N. (2024). Green synthesis of ZnO nanoparticles using Nigella sativa seed extract for antibacterial activities. Nano-Structures Nano-Objects 38, 101212. 10.1016/j.nanoso.2024.101212

[B71] Wu J. Yang W. Song R. Li Z. Jia X. Zhang H. (2023). Dietary soybean lecithin improves growth, immunity, antioxidant capability and intestinal barrier functions in largemouth bass Micropterus salmoides juveniles. Metabolites 13, 512. 10.3390/metabo13040512 37110170 PMC10145076

[B72] Yadav S. Pandey A. Mali S. N. (2024). From lab to nature: recent advancements in the journey of gastroprotective agents from medicinal chemistry to phytotherapy. Eur. J. Med. Chem. 272, 116436. 10.1016/j.ejmech.2024.116436 38704935

[B73] Zambonino M. C. Quizhpe E. M. Mouheb L. Rahman A. Agathos S. N. Dahoumane S. A. (2023). Biogenic selenium nanoparticles in biomedical sciences: properties, current trends, novel opportunities and emerging challenges in Theranostic nanomedicine. Nanomaterials 13, 424. 10.3390/nano13030424 36770385 PMC9921003

[B74] Zheng Y. Sun J. Luo Z. Li Y. Huang Y. (2024). Emerging mechanisms of lipid peroxidation in regulated cell death and its physiological implications. Cell Death Dis. 15, 859–19. 10.1038/s41419-024-07244-x 39587094 PMC11589755

